# Outcomes of pelvic and para-aortic stereotactic reirradiation for gynaecological cancer recurrence

**DOI:** 10.1016/j.ctro.2025.101060

**Published:** 2025-10-18

**Authors:** Benjamin J Thomas, Kallol Bhadra, Ian Zing Tan, Lei Wang, Susan Lalondrelle

**Affiliations:** a The Royal Marsden NHS Foundation Trust, Department of Radiotherapy, Downs Road, Sutton, London SM2 5PT, United Kingdom; b The Institute of Cancer Research, Division of Radiotherapy and Imaging, 15 Cotswold Road, Sutton, London SM2 5NG, United Kingdom

**Keywords:** Reirradiation, Radiotherapy, Stereotactic, Oligometastatic, Pelvic, Para-aortic, Gynaecological, Cervix, Uterine, Vulval, Vaginal, Ovarian

## Abstract

**Introduction:**

Recurrence of gynaecological cancer in the pelvis is associated with significant morbidity and can be challenging to treat, particularly when arising in a previously irradiated site. For cases of oligometastatic relapse a targeted approach is preferable, avoiding the need for systemic therapy. Type 1 reirradiation using highly conformal techniques such as stereotactic radiotherapy (SBRT) is increasingly used, aiming for disease ablation at the site of relapse. We present an analysis of our institutional experience of using SBRT for gynaecological cancer reirradiation to identify efficacy, toxicity and other factors for appropriate case selection.

**Material and methods:**

Patients treated with pelvic or para-aortic SBRT reirradiation at our institution for histologically proven gynaecological malignancies between July 2012 and January 2021 were identified from local records. Indications for treatment included soft tissue and nodal recurrences and cases of positive margin following surgery. Data were collected on survival, failure patterns, demographics, planning and dosimetry.

**Results:**

73 patients were identified who had undergone 81 courses of pelvic or para-aortic reirradiation using SBRT. Median prescribed dose was 30 Gy in 5 fractions. Following SBRT reirradiation, median overall survival was 42.9 months (95 % confidence interval, CI 36.0 to 68.9 months), median progression-free survival was 14.0 months (95 % CI 9.2 to 24.3 months). Median time to local failure of the treated lesion following SBRT reirradiation was 35.2 months (95 % CI 24.3 to not reached). SBRT reirradiation was well tolerated with acute grade 3 toxicity reported in two patients. Late grade 3 toxicity was documented in two patients, both occurring over 2 years following treatment.

**Conclusion:**

In selected patients with oligometastatic pelvic or para-aortic relapse of gynaecological cancer following previous pelvic radiotherapy, SBRT reirradiation is an effective and safe treatment modality. SBRT reirradiation can provide durable local control and delay time to further treatment by more than 12 months.

## Highlights

• Stereotactic reirradiation of pelvic oligometastatic disease is effective.
• Median PFS of 14 months following pelvic or para-aortic SBRT reirradiation.
• Benefits of SBRT reirradiation seen in both cervix and uterine cancer patients.
• Acute and late radiation toxicity is minimal post SBRT reirradiation.

## 1 Introduction

Gynaecological cancers are frequently treated with radiotherapy at the time of initial diagnosis; either in the form of primary radical treatment or in the adjuvant setting, often with the addition of concurrent chemotherapy. When relapse occurs in a metachronous oligometastatic setting [1] a targeted approach is favoured, as this may delay the need for systemic therapy, and in a small percentage of patients potentially offer a curative salvage treatment. Surgery has an established role in this setting [2,3], but often necessitates an exenterative approach to obtain complete resection with clear margins. Frequently the anatomical proximity of critical structures or predicted post-operative morbidity mean this treatment option may not be feasible, especially in the previously irradiated pelvis [4]. Reirradiation is a new course of radiotherapy to a previously irradiated volume. In this clinical setting it refers to reirradiation type 1, where a new course of treatment has geometrical overlap with irradiated volume of previous courses [5], and offers a potential therapeutic alternative.

Stereotactic radiotherapy (SBRT), a highly conformal image-guided radiotherapy technique, has demonstrated improvement in overall survival in oligometastatic disease in the Phase II SABR-COMET trial [6]. In the United Kingdom, SBRT in oligometastatic disease was also investigated in the pelvic reirradiation setting through the single-arm prospective observational Commissioning through Evaluation (CtE) project [7,8], and lead to the approval of this technique in the UK [9].

There are a number of published case series on SBRT reirradiation for oligometastatic relapse in gynaecological cancer [[10], [11], [12], [13], [14], [15], [16], [17], [18], [19], [20]]. However, there is significant heterogeneity amongst the data sets, including mixed cohorts of patients with in-field recurrence, marginal recurrence (often at the para-aortic region), recurrence in patients treated with primary brachytherapy only, and relapse within an unirradiated pelvis or treatment of a distant site. There is also a lack of guidance and agreement on case selection, technique, dose and fractionation [[21], [22], [23]]. We present a retrospective analysis of our institutional experience of using SBRT for gynaecological cancer reirradiation to identify efficacy, toxicity and other factors to further guide appropriate case selection in this setting.

## 2 Material and methods

Following institutional review board approval of this service evaluation, patients treated with pelvic or para-aortic SBRT reirradiation at our institution for histologically proven gynaecological malignancies between July 2012 and January 2021 were identified from local records. Primary sites included cervix, uterine, vaginal, vulval and ovarian cancers. Eligibility criteria for SBRT pelvic or para-aortic reirradiation included lymph node or soft tissue recurrence in the pelvis or para-aortic region, or a positive margin after salvage surgical resection, expected gross tumour volume (GTV) or clinical target volume (CTV) in post-operative cases < 6 cm maximum diameter, 1–3 sites of metastatic relapse, expected life expectancy of > 6 months, no significant ongoing toxicity from previous radiation, ideally > 6 months since initial radiation treatment and WHO Performance Status ≤ 2. Cases were discussed and approved prospectively by institutional multidisciplinary team meeting prior to treatment.

All patients who received prior radiotherapy to the pelvis, with or without para-aortic radiotherapy, were included without mandating a minimum dose. Patients who received adjuvant vaginal vault brachytherapy alone were also included.

Previous treatment plans were rigidly registered with the new planning scan to assess the degree of overlap for relevant organs at risk (OAR). We did not perform deformable registration of previous treatment from DICOM images if these were available. OAR may often have been displaced due to organ mobility or interim surgery, therefore deformable registration was felt to be unreliable for dose estimation. If previous treatment details were available, an estimate of equivalent dose in 2 Gy fractions (EQD2) max dose to each OAR in proximity to the reirradiation site was made and the remaining dose constraints in EQD2 were calculated back to meet published SBRT constraints [24,25]. If the original course of radiotherapy was performed at another centre and the treatment plan was not available, it was assumed that OAR in the vicinity of the new target had received the prescription dose from the original course. Contouring of GTV was done via fused MRI and planning CT scans, and contouring of CTV in cases of post-operative positive margin was aided by surgical operation notes and histology report.

SBRT was delivered on a variety of platforms including C-arm linear accelerator, The Cyberknife® System (Accuray, Sunnyvale, CA, USA), and Elekta Unity MR-linac (Elekta AB, Stockholm, Sweden). Data for analysis was collected from hospital electronic records and radiotherapy treatment planning systems.

We recorded patient and tumour demographic data, including age at diagnosis and at reirradiation, histological subtype, initial staging and performance status. We recorded radiotherapy planning data, including GTV or CTV volume, and treatment site within the pelvis or para-aortic region. Timepoints for disease relapse and death were collated from clinical and radiological reports to calculate the endpoints of overall survival (OS), progression-free survival (PFS) (measured from last clinical or imaging follow-up), local failure (measured from last imaging follow up), locoregional and distant failure, and median follow-up duration, as calculated by reverse Kaplan-Meier approach. Data on acute and late toxicity was collected; where available this was collated from prospective toxicity analysis (CTCAE 4.0 grading) during the CtE study period, however in cases of long-term follow up following study completion or if this was not prospectively recorded, toxicity was inferred from review of patient case notes. Patients were censored at their last recorded follow-up or up to September 2023.

Cox proportional hazards regression model was used to evaluate associations between patient and treatment variables and time to progression. Covariates included in the multivariate model included gynaecological cancer subsite, age at time of reirradiation, time from primary radiotherapy to reirradiation, treatment indication (soft tissue / lymph node / positive margin post-operatively), and GTV/CTV volume. The proportional hazards assumption was tested using Schoenfeld residuals.

Statistical analysis was performed using written code from Python 3.13 in PyCharm CE and Lifeline package, and IBM SPSS Statistics (IBM Corp. Released 2023. IBM SPSS Statistics for Windows, Version 29.0.2.0 Armonk, NY: IBM Corp) for formulating Kaplan-Meier survival curves.

## 3 Results

73 patients were identified who had undergone 81 episodes of SBRT pelvic or para-aortic reirradiation: eight patients received two courses of SBRT (four receiving contemporaneous SBRT to two treatment sites, four receiving sequential SBRT due to disease progression following first SBRT course). Treatment data is included in Table 1. Most of the cohort had cervix or uterine primary cancer with 33 (45.2 %) and 27 (37.0 %) patients respectively. There were smaller numbers for ovarian (6, 8.2 %), vaginal (4, 5.5 %), and vulval (3, 4.1 %) primary sites.

**Table 1:** Treatment Characteristics of patient cohort treated with SBRT pelvic and para-aortic reirradiation.

**Primary gynaecological malignancy n (%)**		
Cervix	33	(45.2 %)
Uterus	27	(37.0 %)
Ovary	6	(8.2 %)
Vagina	4	(5.5 %)
Vulva	3	(4.1 %)
**Primary radiotherapy modality n (%)**		
EBRT + brachytherapy	52	(71.2 %)
EBRT with Phase 2 EBRT boost	6	(8.2 %)
Single phase EBRT only	12	(16.4 %)
Vaginal vault brachytherapy only	3	(4.1 %)
**Primary EBRT dose fractionation**		
Median 45 Gy/25# (Range 24 Gy/12# to 54 Gy/28#)		
**Time from primary radiotherapy to reirradiation**		
Median 28 months (Range 4 months to 30 years)		
**Prescribed SBRT dose**		
Median 30 Gy/5# (Range 24 Gy/3# to 33 Gy/3#)		
**Delivery platform n (%)**		
Cyberknife®	53	(65.4 %)
C-arm linac	22	(27.2 %)
MR-linac	6	(7.4 %)
**Treatment site n (%)**		
Soft tissue	49	(60.5 %)
Lymph node	20	(24.7 %)
Positive surgical margin	12	(14.8 %)
**Treatment location n (%)**		
Pelvis	71	(87.7 %)
Para-aortic region	10	(12.3 %)
**GTV volume**		
Median = 16.3 cc (Range 2.1 to 91.8 cc)		
**CTV volume (for treatment of positive surgical margin)**		
Median = 27.4 cc (Range 8.1 to 66.4 cc)		

All patients received previous radiotherapy: 70 (95.9 %) had external beam radiotherapy (EBRT); 52 (71.2 %) had both EBRT and intrauterine or vaginal vault brachytherapy, 6 (8.2 %) had EBRT Phase 2 boost, 12 (16.4 %) had single phase EBRT. 3 (4.1 %) patients had adjuvant vaginal vault brachytherapy alone. The median EBRT dose-fractionation was 45 Gy/25# (Range 24 Gy/12# to 54 Gy/28#), with additional numerical breakdown of primary dose-fractionation across the whole cohort, alongside breakdown of brachytherapy component supplied within Supplementary material Table S1, although as brachytherapy doses from other centres was not readily available; brachytherapy doses and fractionation are not described. Median time from primary radiotherapy to reirradiation was 28 months (Range 4 months to 30 years).

Treatment indication was varied with 49 (67.1 %) soft tissue sites, 20 (27.2 %) lymph node sites, and 12 (14.8 %) positive surgical margins post-operatively. Median size of GTV was 16.3 cubic centimetre (cc) (Range 2.1 to 91.8 cc) and CTV was 27.4 cc (Range 8.1 to 66.4 cc). Median prescription dose was 30 Gy/5# delivered on an alternate day basis, adapted to dose constraints if this was necessary. Most patients were treated on the Cyberknife® (65.4 %) platform, followed by C-arm linac (27.2 %) and MR-Linac (7.4 %). Median follow-up duration was 60.5 months (95 % confidence interval 38.7 to 67.9 months).

**Fig. 1:**
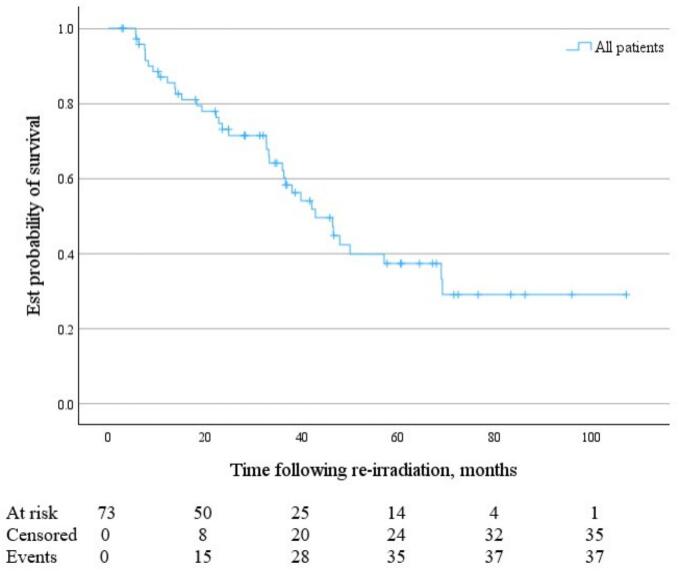
KM Curve of OS following pelvic and para-aortic SBRT reirradiation for gynaecological malignancies.

**Fig. 2:**
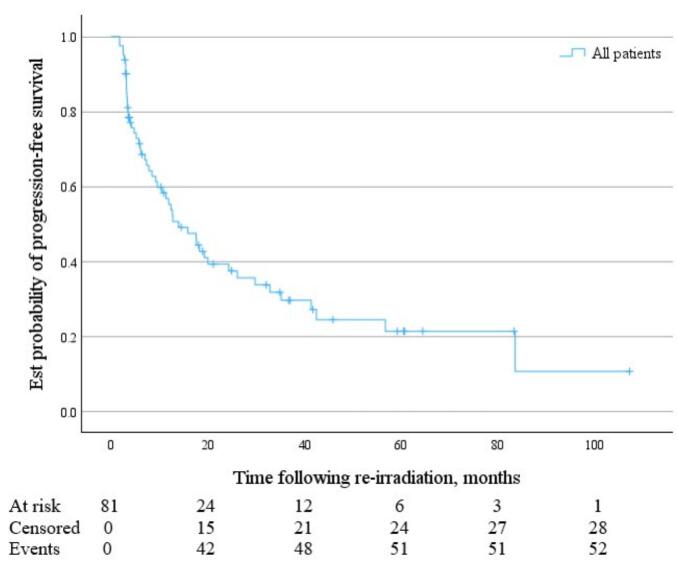
KM Curve of PFS following pelvic and para-aortic SBRT reirradiation for gynaecological malignancies.

**Fig. 3:**
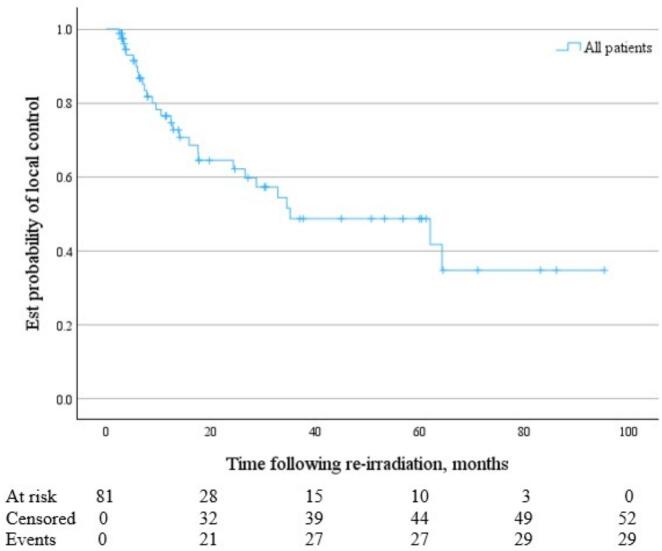
KM Curve of time to local failure following pelvic and para-aortic SBRT reirradiation for gynaecological malignancies.

Following SBRT reirradiation, grouped median OS was 42.9 months (95 % CI 36.0 to 68.9 months) (Fig. 1), and median PFS was 14.0 months (95 % CI 9.2 to 24.3 months) (Fig. 2). Median time to local failure of the treated lesion following SBRT reirradiation was 35.2 months (95 % CI 24.3 months to not reached) (Fig. 3).

**Fig. 4:**
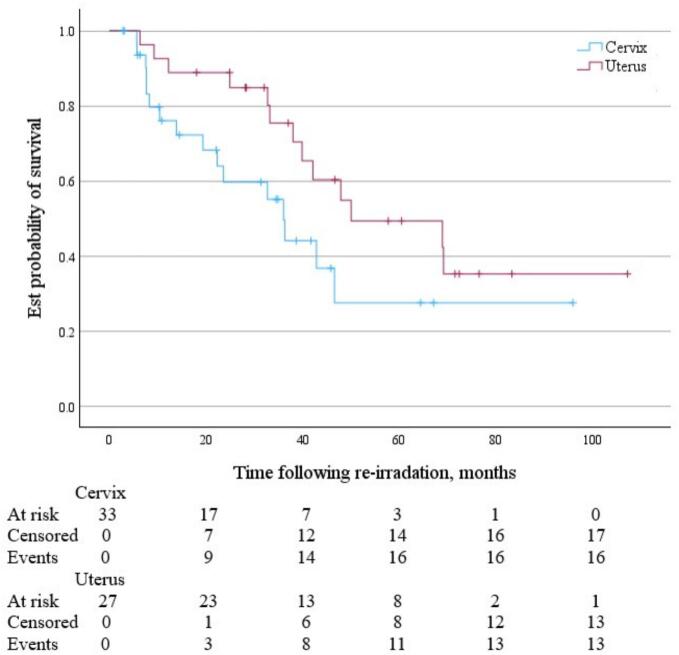
KM Curve of OS following pelvic and para-aortic SBRT reirradiation for cervix (blue) and uterine (red) malignancies. (For interpretation of the references to colour in this figure legend, the reader is referred to the web version of this article.)

**Fig. 5:**
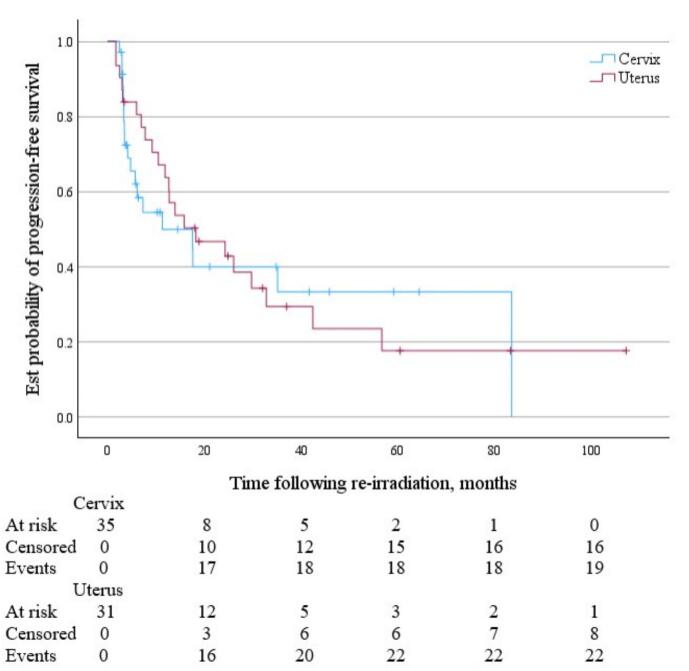
KM Curve of PFS following pelvic and para-aortic SBRT reirradiation for cervix (blue) and uterine (red) malignancies. (For interpretation of the references to colour in this figure legend, the reader is referred to the web version of this article.)

**Fig. 6:**
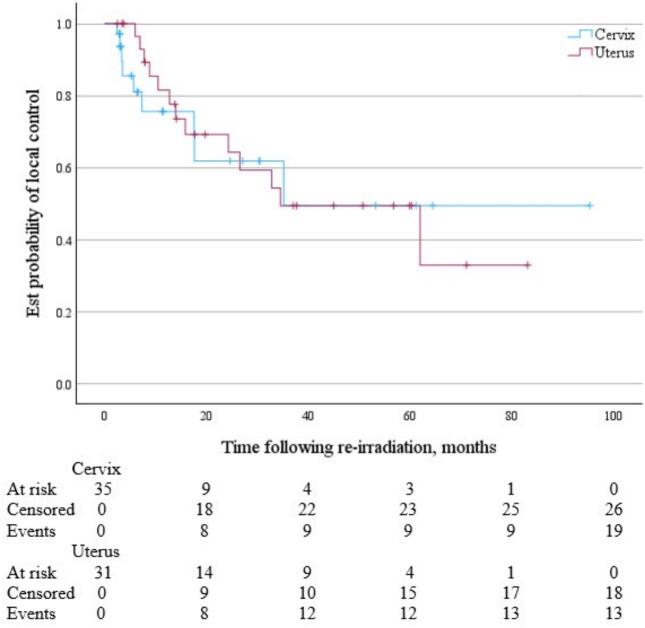
KM Curve of time to local failure following pelvic and para-aortic SBRT reirradiation for cervix (blue) and uterine (red) malignancies. (For interpretation of the references to colour in this figure legend, the reader is referred to the web version of this article.)

For cervix and uterine cancer patients, following SBRT reirradiation the median OS was 36.0 months (95 % CI 19.4 months to not reached) and 50.0 months (95 % CI 38.0 months to not reached) respectively *(*Fig. 4*)*. Median PFS was 11.3 months (95 % CI 4.2 to 83.6 months) and 18.3 months (95 % CI 10.5 to 32.8 months) respectively (Fig. 5). Median time to local failure of the treated lesion following SBRT reirradiation was 35.2 months (95 % CI 17.6 months to not reached) for cervix cancer and 34.5 months (95 % CI 15.9 months to not reached) for uterine cancer (Fig. 6).

SBRT reirradiation was well tolerated with acute grade 3 toxicity reported in two patients (diarrhoea and fatigue, respectively). Late grade 3 toxicity was documented in two patients (nausea and vaginal stricture, respectively), both occurring over 2 years following treatment.

No covariates (i.e. gynaecological cancer subsite, age at time of reirradiation, time from primary radiotherapy to reirradiation, treatment indication, GTV/CTV volume) reached statistical significance at the 0.05 level, on multivariate Cox proportional hazards analysis assessing factors associated with progression following SBRT reirradiation. Vulval cancer did show a non statistically-significant trend towards risk of progression (HR 2.63, 95 % CI 0.81 – 8.58, p = 0.108). Results of multivariate analysis are included in Supplementary material Table S2.

## 4 Discussion

Our data demonstrates that in cases of oligometastatic pelvic and para-aortic relapse of gynaecological cancer following previous pelvic radiotherapy, SBRT reirradiation is an effective and safe treatment modality. Our series consists of 73 patients, all of whom had received prior radiation and were treated with defined inclusion criteria and consistent dose fractionation schedules. Whilst the patients are a selected cohort with a lower burden of metastatic disease at the time of reirradiation; our grouped median overall survival of over 3.5 years (42.9 months) demonstrate that with appropriate case selection SBRT reirradiation may extend survival in relapsed disease. These results for overall survival are similar to other published series of SBRT reirradiation in gynaecological cancer: Park et al presented a series of 85 patients, of whom 83.5 % had received previous radiotherapy, reporting a median OS of 32.7 months [14], and Ling et al described a series of 20 patients, all of whom had previous radiotherapy, and reported a median OS of 64.5 months [16]. Due to heterogeneity of case selection within these cohorts and our own, direct comparison is not appropriate, but it can be remarked generally that these survival rates are extended compared to historical outcomes for The International Federation of Gynaecology and Obstetrics (FIGO) Stage IV disease in cervix, uterine, vulval and ovarian cancer. Grouped median PFS of over 12 months reinforces the potential of SBRT reirradiation for delaying requirements for further therapy in those with oligometastatic disease, including the potential for sequential treatment to further sites of metachronous oligometastatic disease. In our cohort, none of the patients had a second course of SBRT to the same lesion however.

Outcomes differed according to primary tumour site, but there was consistent response in OS and PFS for cervix and uterine cancers from SBRT reirradiation. These benefits were more pronounced in uterine cancers with longer PFS and OS, although in both groups median overall survival was multiple years following relapsed disease. This is particularly promising as many of these patients were treated prior to the availability of immunotherapy and small molecule inhibitors as a treatment option in metastatic disease. Arguably, the lower burden of disease in this oligometastatic population would correspond to more favourable outcomes, however, the benefit of SBRT reirradiation is seen across histologies, although some differences in PFS may be explained by differing histologies and tumour biology between cervix and uterine cancer. In addition, these cohorts are smaller in number individually, with wider confidence intervals, and as such it has to be acknowledged there is more uncertainty regarding benefits in these subgroups, although our data is similar to other smaller case series which analysed cervix or uterine cancer individually.

Our data also demonstrate that SBRT reirradiation was generally well tolerated, with minimal acute and late toxicity. We acknowledge that follow-up data from external institutions was not always available and may have led to under-reporting of late effects. In addition, in some cases toxicity was not formally prospectively recorded, and as such had to be inferred from review of case notes, particularly for long-term follow up. Therefore, these results do need to be interpreted with a degree of caution. However, other studies support the tolerability of SBRT. The UK CtE study [7], which included reirradiation of pelvic relapse from all (including non-gynaecological) tumour types reported similar low levels of grade 3 toxicity at 3.8 % [8]. Pooled data in SBRT reirradiation in the pelvis (again not limited to gynaecological malignancies) reported grade three or four toxicity rate estimation of 6.3 % [26]. These studies, combined with our own case series, do demonstrate SBRT reirradiation in the pelvis and para-aortic region to be well tolerated, however further work is needed to gain sufficient understanding of late toxicities.

There are other limitations in this work. There is heterogeneity in our data set with inclusion of a range of primary gynaecological malignancies with differing biology, primary radiotherapy modalities (although 96 % of patients did receive EBRT to the pelvis at a minimum), and varying treatment targets including soft tissue or lymph node recurrence and positive surgical margins. It would be challenging however to assemble a more homogenous dataset with sufficient patient numbers to draw more robust conclusions without multi-centre collaboration. Although multivariate Cox proportional hazards analysis did not demonstrate any statistically significant association of patient and tumour characteristics with progression following SBRT-reirradiation, there was a non-significant trend towards worse outcomes from vulval cancer. However, it is important to note that this was not statistically significant and case numbers outside of uterine and cervix cancer were low and therefore these results need to be interpreted with a substantial degree of caution; further multi-centre prospective data collection in rarer cancers, such as vulval cancer, may help to analyse this further. Deformable image registration was not reliably available and a significant number of our patients received their primary radiotherapy at a different radiotherapy treatment centre, so previous dosimetric data was not always available and the degree of overlap may have been overestimated.

Although the local failure rate was modest, with median time to local failure of the treated lesion of just under 3 years, this is comparable to other case series with either whole cohorts of reirradiation (albeit with some possible marginal recurrences) or those who reported reirradiation cases separately: Ling et al reported a 3-year in field local control rate of 61.4 % [16], and Park et al reported local PFS of 60.2 % at 2 years among reirradiation patients [14]. We also have to acknowledge that our local failure data is the most heavily censored, and so we need to be cautious in terms of interpretation of this data. Possible reasons for local failure include primary or acquired radioresistance, insufficient dose and inaccurate target coverage. Regarding dose fractionation, in our series this was conservative and generally not escalated beyond 30 Gy in 5 fractions, even if OAR constraints were comfortably met. Other case series have reported a dose–response relationship with increased BED corresponding to improved local control [14,16,19], and it may have been that some patients would have benefited from further dose escalation. As safety of SBRT reirradiation has been demonstrated, further work on iso-toxic dose escalation could improve disease control to provide a durable salvage treatment. There is limited published data on this in gynaecological SBRT reirradiation, but principles from published offline work in SBRT reirradiation in rectal cancer could inform further work in this area [27].

SBRT for pelvic reirradiation is being increasingly adopted, but with a heterogeneity of techniques, doses and indications. International consensus documents from ESTRO/EORTC [5] are helpful to standardise therapy guidelines and compare outcomes more easily, given that randomised controlled trials and tumour specific studies are unlikely to be financially viable. Further work is also needed to establish protocols for accounting for normal tissue recovery following initial radiotherapy. There is currently a lack of published data and consensus on whether to account for recovery in the reirradiation setting [21,22], however a pragmatic approach of factoring in a percentage of recovery, particularly when reirradiation occurs greater than 18 months after initial radiotherapy can be considered [26]. UK SABR Consortium Guidance cautiously suggests 30 % recovery, rising to 50 % in those with an interval greater than 18 months from primary radiotherapy [28]. However, it is recognised that this is a cautious non-evidence based pragmatic approach in incorporating recovery, and partially related to a survey of clinicians participating in the wider CtE project. Therefore, the degree to which recovery is incorporated, if at all, needs to be individualised to the patient. Further data collection on reirradiation dose, efficacy and toxicity through national audit [28] and international collaboration through work such as the ReCare (EORTC 2011-RP) study [29] will hopefully provide further real-world evidence in this area in future, alongside the DARIUS study [30], which is aiming to gain consensus on dose accumulation and recovery in relation to toxicity. There is also increasing data regarding safety and efficacy of other reirradiation techniques such as brachytherapy [[31], [32], [33]], and proton beam radiotherapy [34], alongside established surgical techniques such as pelvic exenteration; appropriate case selection for salvage treatment strategy including consideration of suitable reirradiation modality, taking into account local expertise, will likely be key for improving patient outcomes in oligometastatic relapse of gynaecological malignancies.

## 5 Conclusion

SBRT reirradiation is a safe and effective therapy for the management of oligometastatic relapse in the pelvic and para-aortic region for patients with gynaecological cancers. We observed durable local control and delayed time to further therapy by over 12 months, with benefits seen across histologies. Prospective standardised collaborative data collection in future will provide further understanding of the most appropriate patient selection criteria and optimal dose fractionation.

## Declaration of Competing Interest

The authors declare the following financial interests/personal relationships which may be considered as potential competing interests: Susan Lalondrelle reports a relationship with Elekta AB that includes: travel reimbursement. Susan Lalondrelle reports a relationship with Accuray Inc that includes: speaking and lecture fees. If there are other authors, they declare that they have no known competing financial interests or personal relationships that could have appeared to influence the work reported in this paper.
