# Supplementary material for: Outcomes of pelvic and para-aortic stereotactic reirradiation for gynaecological cancer recurrence
Source: Clin Transl Radiat Oncol. 2025 Oct 18;56:101060. doi: 10.1016/j.ctro.2025.101060 (PMC12593702; doi:10.1016/j.ctro.2025.101060)
Supplement: Supplementary Data 1 [file mmc1.docx]

**Supplementary Material – Table S1**

| **Primary EBRT dose-fractionation and Brachytherapy Modality Breakdown**  **Primary EBRT dose fractionation breakdown n (%)**  45Gy/25# 47 (67.1%)  50.4Gy/28# 13 (18.6%)  30Gy/10# 2 (2.9%)  56Gy/28# 1 (1.4%)  40Gy/15# 1 (1.4%)  46.8Gy/26# (Stopped early due to toxicity) 1 (1.4%)  37.8Gy/21# (Stopped early due to toxicity) 1 (1.4%)  30Gy/6# 1 (1.4%)  24Gy/12# (Plus brachytherapy 22Gy/4#) 1 (1.4%)  Not numerically recorded in case notes 2 (2.9%)  1x Cervix – Radical CRT with intrauterine brachytherapy  1x Endometrium – Post-op EBRT with vaginal vault brachytherapy  **Primary Brachytherapy modality breakdown n (%)**  Intrauterine brachytherapy 27 (49.1%)  Vaginal vault brachytherapy boost 25 (45.4%)  Vaginal vault brachytherapy alone 3 (5.5%) |
| --- |

*Table S1*. Primary EBRT dose-fractionation and brachytherapy modality breakdown
