# Supplementary material for: Outcomes of pelvic and para-aortic stereotactic reirradiation for gynaecological cancer recurrence
Source: Clin Transl Radiat Oncol. 2025 Oct 18;56:101060. doi: 10.1016/j.ctro.2025.101060 (PMC12593702; doi:10.1016/j.ctro.2025.101060)
Supplement: Supplementary Data 2 [file mmc2.docx]

**Supplementary Material – Table S2**

| **Multivariate Cox Proportional Hazards Model for progression following SBRT reirradiation**  **Variable Hazard Ratio (HR) 95% CI p-value** Age at Reirradiation 1.01 0.98 – 1.03 0.668 Time to Reirradiation 1.00 0.99 – 1.00 0.909 Tumour subsite  Uterine Reference  Cervix 1.25 0.54 – 2.88 0.606  Ovary 1.67 0.65 – 4.30 0.283  Vulva 2.63 0.81 – 8.58 0.108  Vaginal 0.43 0.04 – 4.52 0.484 Treatment site  Soft tissue Reference  Lymph Node 1.30 0.54 – 3.17 0.560  Positive margin 1.07 0.46 – 2.50 0.880 GTV/CTV volume 0.86 0.43 – 1.72 0.672 |
| --- |

*Table S2*. Multivariate Cox Proportional Hazards Model for progression following SBRT reirradiation

Tests of the proportional hazards assumption using Schoenfeld residuals indicated no violations for any covariate or the model globally (global test: *p* = 0.76).
